# Factors affecting family consent to corneal donation at a hospital-based eye bank in a tertiary Israeli medical center

**DOI:** 10.1371/journal.pone.0357908

**Published:** 2026-09-17

**Authors:** Eden Amir, Netanel Corem, Dean David Lichter, Tamar Ashkenazi, Abraham Solomon

**Affiliations:** 1 Department of Ophthalmology, Hadassah-Hebrew University Medical Center, Jerusalem, Israel; 2 Department of Military Medicine and "Tzameret", Faculty of Medicine, The Hebrew University of Jerusalem and Israel Defense Forces Medical Corps, Jerusalem, Israel; 3 Israel National Transplant Center, Israel Ministry of Health, Tel Aviv, Israel; Cardiff Metropolitan University, UNITED KINGDOM OF GREAT BRITAIN AND NORTHERN IRELAND

## Abstract

**Purpose:**

To identify clinical, demographic, and situational factors associated with family consent to corneal donation in a culturally diverse Israeli medical center operating under an opt-in system.

**Methods:**

This retrospective observational study included 824 deceased individuals eligible for corneal donation at Hadassah Medical Center between 2019 and 2022. Data were collected on various variables, such as religion, Jewish religiosity, donor card registration, hospitalization details, method of family approach (face-to-face or telephone), next of kin identity and relation to consent for corneal donation. Univariate and multivariate analyses were performed to identify factors associated with family consent to corneal donation.

**Results:**

Overall, 30.7% of families consented to corneal donation. In multivariable analyses, older age, religion, Jewish religiosity, donor card registration of the deceased, and donor card registration of a first-degree family member were independently associated with family consent. Jewish families were more likely to consent than non-Jewish families (OR=7.79), and within the Jewish population, secular and modern Orthodox families had the highest consent rates (57.3% and 53.6%, respectively). Donor card registration showed the strongest association with consent, with the highest consent rate observed when both the deceased and a first-degree family member were registered (83.3%, OR = 29.80). Consent rates did not differ significantly between telephone and face-to-face approaches.

**Conclusions:**

Religion, Jewish religiosity, and donor card registration were independently associated with family consent to corneal donation. Comparable consent rates following telephone and face-to-face approaches suggest that telephone based requests may be an effective alternative when in-person discussions are not feasible. These findings support efforts to increase awareness of corneal donation, promote donor card registration, and tailor communication strategies to different cultural and religious communities.

## Introduction

The cornea is the transparent front layer of the eye, functioning as the window through which light enters. Damage or distortion of the cornea can interfere with the transmission of light and lead to visual impairment or even blindness.

According to a World Health Organization (WHO) survey, corneal disease and injury constitute the third leading cause of blindness worldwide [1]. This type of blindness is often reversible through corneal transplantation. It is estimated that over ten million people globally are currently in need of such procedures [2].

Corneal transplantation involves partial or full replacement of a damaged cornea with tissue from a deceased human donor [3]. Corneal tissue may be recovered either by whole eye enucleation followed by eye bank processing, or by in situ excision of a corneoscleral button. At Hadassah Medical Center, corneal tissue is routinely recovered using the latter technique. Corneal transplantation has high success rates, depending on the type of surgery and clinical indication [3].

Approximately 1100 corneal transplants are performed annually in Israel, yet the number of patients awaiting surgery exceeds the number of available donor corneas [4]. Israel follows an opt-in donation policy, whereby individuals can express their willingness to donate organs or tissues after death by signing the Israeli national donor card, known as “ADI” [5]. Signatories are registered in a confidential national database, and the registration can be revoked at any time. Although donor card registration has legal validity, consent from the deceased’s family is still required in practice [6]. As a result, the family’s decision remains a key determinant of donor tissue availability. Despite the availability of many medically eligible deceased individuals, the potential for donation remains underutilized, highlighting family consent as a key challenge in increasing corneal transplantation rates.

The overall corneal supply depends not only on family consent but also on the suitability of the retrieved tissue for transplantation. Endothelial cell density (ECD) is a pivotal criterion that provides a quantifiable measure of corneal tissue health, accounting for a substantial portion of disqualifications for transplantation. Previous work from our group at the same eye bank observed a trend toward higher ECD adequacy with shorter death to retrieval times, even within relatively brief intervals, and confirmed advanced donor age and prior cataract surgery as the strongest predictors of lower ECD [7].

Studies conducted worldwide have identified associations between donor-related characteristics and family consent to corneal donation, including the identity and relationship of the next of kin, cause of death, hospital department, method of approach, and religion [8–12].

Religion has been recognized as an important factor influencing attitudes toward organ and tissue donation worldwide. Although Judaism, Islam, and Christianity generally support organ and tissue donation, previous studies have shown that religious affiliation and the degree of religiosity may influence donation decisions through differences in religious interpretation and cultural practices [13–16].

Given Israel’s unique religious diversity and previous evidence that religiosity influences donation attitudes within the Jewish population, religion and Jewish religiosity were therefore included among the variables evaluated for their association with family consent to corneal donation.

Notably, most Israeli studies have focused on organ donation rather than corneal donation [17]. The key distinction is that corneal tissue is typically retrieved after cardiac death, whereas solid organ donation generally requires brain death. In Israel, the definition of brain death remains the subject of cultural and religious debate [18]. Therefore, findings from studies of organ donation may not fully apply to the setting of corneal donation.

The eye bank at Hadassah Medical Center is one of the largest eye banks in Israel. The hospital serves a culturally and religiously diverse population, and the eye bank regularly interacts with families across a broad spectrum of religious observance. This diversity presents both challenges and opportunities in obtaining family consent. Eye banks in Israel also distinguish themselves with the short timing of corneal donation requests after death, attributed to the location of eye banks within hospital premises and religious considerations rooted in Jewish tradition regarding burial with minimal delay.

Understanding the characteristics associated with family consent can inform more effective and culturally sensitive approaches to donation requests. By identifying factors linked to higher or lower likelihoods of consent, strategies can be refined to improve consent rates and better match donor availability with clinical need.

This study uniquely examines a broad range of clinical, demographic, situational, and cultural factors to better understand what influences family consent to corneal donation.

## Methods

### Study design and population

This retrospective observational study was conducted at Hadassah Medical Center between 2019 and 2022. Recorded family approaches regarding consent for corneal donation were analyzed. Of the 3087 patients who died during the study period, 824 (26.7%) were considered eligible for approach based on medical and procedural criteria.

Inclusion criteria included patients aged 5–80 years without medical contraindications for corneal donation, as defined by Ministry of Health guidelines [19]. Exclusion criteria included, but were not limited to, resistant bloodstream infections, positive serology for HIV, HBV, or HCV, hematologic malignancies, diseases affecting the cornea, and cases in which the family could not be contacted or hastened to bury the deceased.

### Data collection

Most family approaches (approximately 85%) were conducted by a dedicated corneal transplant coordination team composed of three coordinators. The remaining requests were carried out by nursing staff in the respective hospital departments.

After each approach, coordinators completed a standardized Ministry of Health form documenting the family’s decision, the identity and relationship of the decision maker, the method of contact (face-to-face or telephone), and the hospital department. Additional information, including the time interval between death and the approach, the deceased’s religion, and, for Jewish families, an assessment of religiosity, was recorded internally by the transplant coordination team.

Participants were classified into four groups: secular, traditional, modern orthodox, and ultra-Orthodox. In Israel, religiosity can represent membership in distinct socioreligious subgroups rather than as a purely individual level characteristic. These subgroups are characterized by differences in outward appearance, language use, and belief systems, which are often externally identifiable. Secular Jews typically wear contemporary Western style clothing and generally do not display prominent religious symbols in daily life. Traditional Jews often dress similarly to secular Jews but may incorporate selective religious practices or symbols, and their conversations may still include references to religion. Modern orthodox Jews usually combine modern dress with visible religious observance, and their speech may more often include explicitly religious themes. Among ultra-Orthodox Jews, distinctive dress is common, and decisions regarding medical care may be guided by rabbinic consultation [17,20,21]. Therefore, religiosity was determined based on observable indicators and the coordinator’s impression during the conversation.

Clinical and demographic data, including cause of death, the duration of the final hospitalization, and the total number of hospitalization days in the last year of life, were extracted from the hospital’s electronic medical records. Causes of death were categorized based on the official death certificate and relevant clinical documentation.

Information regarding donor card registration of the deceased and their first-degree relatives was obtained from the National Organ Donor Card Registry of the Israeli Ministry of Health and securely linked to the study dataset.

### Ethical considerations

The study was approved by the Institutional Review Board of Hadassah Medical Center (Approval No. 0708–22-HMO) on 20^th^ February 2023 and conducted in accordance with the Declaration of Helsinki. The data in this study were accessed for research on 1st March 2023. Prior to conducting data analysis, identifiable participant information was anonymized.

### Variables

Demographic variables included age (as a continuous variable and in four categories: ≤ 24, 25–44, 45–65, > 65 as in the study of Lee et al. [12]), sex, and marital status. Clinical variables included cause of death and the hospital department at the time of death. Religious variables included religion (Jewish, Muslim, Christian) and, for Jewish participants, religiosity (secular, traditional, modern Orthodox, ultra-Orthodox).

Situational factors included the day and time of death, nurse shift (morning, evening, night), relationship of the next of kin to the deceased, method of contact (face-to-face or telephone), and time from death to request. Two additional variables included the duration of the last hospitalization and the total number of hospitalization days in the last year of life. Donor card registration was recorded using two distinct variables: one indicating whether the deceased was registered, and the other indicating whether a first-degree relative was registered. The primary outcome was family consent to corneal donation (yes/no).

### Statistical analysis

Statistical calculations were performed using SPSS version 28, and RStudio version 4.5. For comparison of quantitative variables between two independent groups, the t-test was used. The relationship between two categorical variables was examined using the Chi-square test (χ2) or Fisher’s exact test. Univariate analysis using a logistic regression model was conducted to evaluate the relationship between consent and various variables. The odds ratio was adjusted to sex and age. A multivariable logistic regression model using the forward stepwise likelihood ratio method was used to simultaneously examine the effect of several variables on the dependent variable (consent). Multivariable analysis was performed only in cases without missing data. All statistical tests were two-tailed, and a P-value of 0.05 or less was considered statistically significant.

## Results

### Characteristics of the deceased

The study population included 824 deceased individuals who were eligible for corneal donation. The average age of the deceased was 63 years, with a median of 67 years and a range between 5 and 80 years. Among them, 462 were male (56.1%) and 362 were female (43.9%). Most of the deceased were married (73.7%). Cancer was the leading cause of death, accounting for 52.2% of cases, followed by infections (14.2%) and cardiovascular conditions (10.4%). At the time of death, most patients were hospitalized in the Oncology department (37.6%), followed by the Internal Medicine department (26.3%) and the Intensive Care Unit (23.8%). In terms of religion, 80.1% were Jewish, 17.0% were Muslims and 2.6% were Christians. In 10 cases religion was not recorded. Among the Jewish group, religious affiliation was documented in 65% of cases. Within this subgroup, 53% were secular, 26.6% ultra-Orthodox, 13.2% modern Orthodox, and 7% traditional.

The mean length of the last hospitalization was 8.3 days, and the mean number of hospitalization days in the last year of life was 32.5 days.

### Characteristics of the next-of-kin

In 78.4% of cases, the identity of the next-of-kin involved in the corneal donation decision was documented. Most decisions were made by the deceased’s children (50.9%) or spouses (29.4%). Other decision-makers included siblings (11.9%), parents (5.9%), and a small group of others (1.9%).

### Donor card registration status

Out of 824 cases, 75 (9.1%) of the deceased were registered as donor card holders. Among these cases, consent was obtained at 77.3%, compared with 26.0% among those who were not registered. In 37 cases (4.5%) of the 824 eligible cases, no next-of-kin was identified in the National Organ Donor Card Registry. Consequently, information regarding next-of-kin donor card registration was available for 787 cases. Of these, 34.8% had a next of kin who was registered as a donor card holder, with a consent rate of 59.9%, compared with 16.2% among those whose next of kin was not registered. When both deceased and kin were registered, consent was highest (83.3%) and lowest when neither were signed (15.0%).

In a subgroup analysis stratified by religion, among Muslims, one individual (0.7%) was registered as a donor card holder, and six cases (4.8%) involved a family member registered as a donor card holder. Among Christians, one individual (4.8%) was registered as a donor card holder, and two cases (13%) involved a family member with a donor card. Among Jews, 73 individuals (11%) were registered as donor card holders, and 264 cases (41%) involved a family member who was registered as a donor card holder.

### Consent rates, methods of contact, and timing

Among the 824 eligible cases, consent for corneal donation was obtained in 30.7% of families. Regarding the method of contact, 63.4% of the requests were made face-to-face, while 36.6% were conducted via telephone. The average time from death to the donation request was 107 minutes, with a median of 82 minutes.

Table 1 summarizes the demographic and characteristics of the study population.

**Table 1:** Demographic and characteristics of the study population.

Category	n (%)	Category	n (%)
**Age, categorized (N = 824)**		**Religion (N = 814)**	
Age <= 24	33 (4.0%)	Jewish	652 (80.1%)
24 < Age <= 44	62 (7.5%)	Muslim	138 (17.0%)
44 < Age <= 65	265 (32.2%)	Christian	21 (2.6%)
Age > 65	464 (56.3%)	Other	3 (0.4%)
**Sex (N = 824)**		**Jewish religiosity (N = 424)**	
Male	462 (56.1%)	Secular	225 (53.1%)
Female	362 (43.9%)	Modern Orthodox	56 (13.2%)
**Marital status (N = 820)**		Ultra-Orthodox	113 (26.7%)
Single	109 (13.3%)	Traditional	30 (7.1%)
Married	604 (73.7%)	**Next of kin deciding (N = 646)**	
Divorced	53 (6.5%)	Spouse	190 (29.4%)
Widow/er	54 (6.6%)	Child	329 (50.9%)
**Cause of death (N = 824)**		Parents	38 (5.9%)
Cardiovascular	86 (10.4%)	Siblings	77 (11.9%)
Cerebrovascular	63 (7.6%)	Other	12 (1.9%)
Respiratory/Organ failure	67 (8.1%)	**Way of contacting (N = 653)**	
Trauma/Injury	48 (5.8%)	Face-to-face	414 (63.4%)
Cancer/Hematology	430 (52.2%)	Telephone	239 (36.6%)
Septicemia/Infection	117 (14.2%)	**Day of the week (N = 824)**	
Other	13 (1.6%)	Sunday	142 (17.2%)
**Hospitalization department (N = 824)**		Monday	115 (14%)
Internal	217 (26.3%)	Tuesday	120 (14.6%)
Surgery	22 (2.7%)	Wednesday	118 (14.3%)
Intensive care units	196 (23.8%)	Thursday	100 (12.1%)
Emergency Department	67 (8.1%)	Friday	105 (12.7%)
Oncology	310 (37.6%)	Saturday	124 (15%)
Hospice	12 (1.5%)	**Donor card registration – deceased (N = 824)**	
**Combined donor card registration (N = 787)**		Yes	75 (9.1%)
Deceased Yes & family Yes	60 (7.6%)	No	749 (90.9%)
Deceased Yes & family No	12 (1.5%)	**Donor card registration – family member (N = 787)**	
Deceased No & family Yes	214 (27.2%)	Yes	274 (34.8%)
Deceased No & family No	501 (63.7%)	No	513 (65.2%)

### Factors affecting consent

Religion and religiosity were strongly associated with consent. Consent was obtained in 240 of 652 Jewish families (36.8%), 5 of 138 Muslim families (3.6%) and 5 of 21 Christian families (23.8%). Overall, Jewish families were significantly more likely to consent than non-Jewish families (OR = 7.79, p < 0.001). Within the Jewish subgroup, Secular and modern Orthodox families showed the highest consent rates (57.3% and 53.6%, respectively), whereas traditional and ultra-Orthodox families had lower consent rates (16.7% and 0.9%, respectively).

The identity of the next-of-kin also influenced consent likelihood, with higher odds observed when decisions were made by parents (OR = 2.96, p < 0.05) or spouses (OR = 1.89, p < 0.05) compared to siblings, who served as the reference group. Department of hospitalization also played a role. Consent was more common among families of patients who died in the Emergency Department (OR = 3.16, p < 0.001), and less likely in Internal Medicine departments (OR = 0.87, p = 0.486).

Donor card registration was a major predictor of consent. Registration of the deceased was associated with markedly higher odds of consent (OR = 10.10, p < 0.001). Registration of at least one first-degree family member was also associated with higher odds of consent (OR = 7.67, p < 0.001). The highest odds of consent were observed when both the deceased and at least one first-degree family member were registered (OR = 29.80, p < 0.001), compared with cases in which neither was registered. Notably, in 17 cases, the deceased were registered as a donor, yet the family declined donation. In 10 of these cases, both the deceased and a family member were registered organ donors, nevertheless, the family declined donation.

There were no significant differences in consent rates between face-to-face and telephone requests (p = 0.800). Consent rates did vary significantly across specific days of the week (p = 0.039), with the lowest rate observed on Fridays (21.9%). However, no significant difference was found between weekends (Friday and Saturday) and weekdays (p = 0.287).

Age showed modest association in its continuous form (p < 0.05) with a mean age of 64.8 years among consents vs. 62.2 years among non-consenting, but no significant association was found when age was categorized (p = 0.127). Other variables, such as sex, cause of death, marital status and nurse shifts, were not significantly associated with consent.

Length of hospitalization showed mixed associations with consent. While the average length of stay at the time of death was not significantly different between groups (p = 0.674), the total number of hospitalization days in the last year was significantly higher among non-consenting families, with a mean difference of 8.7 days (p < 0.01, mean 35.26 vs. 26.50 days).

Table 2 summarizes the association between all tested variables and consent status, including significance levels.

**Table 2:** Summary of univariate associations with consent.

Variable	P value
Religion	p < 0.001*
Jewish religiosity	p < 0.001*
Hospitalization department	p < 0.001*
Donor card registration (deceased)	p < 0.001*
Donor card registration (family member)	p < 0.001*
Hospitalization days in the last year of life	p < 0.01*
Age	p=0.014*
Next of kin	p=0.036*
Day of the week	p=0.039*
Age categorized	p = 0.127
Weekday vs weekend^	p = 0.287
Cause of death	p = 0.306
Sex	p = 0.349
Nurse shifts	p = 0.674
Length of last hospitalization	p = 0.674
Telephone vs face-to-face	p = 0.800
Marital status	p = 0.843

*Factors statistically significant. ^Weekend – Friday and Saturday.

Table 3 presents the results of the univariate analysis conducted on variables that were found significant.

**Table 3:** Univariate analysis of factors affecting consent.

Variable	N	Consent rate, n (%)	Adjusted OR (95% CI)	P value
**Age (years)**	824	**–**	1.01 (1.00-1.02)	P < 0.05
**Religion**	814			p < 0.001
Jewish	652	240 (36.8)	7.79 (4.31-15.6)	p < 0.001
Non-Jewish*	162	11 (6.7)	1.00	
**Religion**	814			p < 0.001
Jewish	652	240 (36.8)	15.15 (6.09-37.67)	p < 0.001
Christian	21	5 (23.8)	8.18 (2.13-31.48)	p < 0.005
Other	3	1 (33.3)	14.66 (1.12-191.66)	p < 0.05
Muslim*	138	5 (3.6)	1.00	
**Jewish religiosity**	424			p < 0.001
Secular	225	129 (57.3)	6.57 (2.42-17.86)	p < 0.001
Modern Orthodox	56	30 (53.6)	5.87 (1.96-17.63)	p < 0.005
Ultra-Orthodox	113	1 (0.9)	0.04 (0.01-0.39)	p < 0.005
Traditional*	30	5 (16.7)	1.00	
**Hospitalization department**	824			p < 0.001
Internal	217	57 (26.3)	0.87 (0.58-1.30)	p = 0.486
Surgery	22	7 (31.8)	1.24 (0.49-3.16)	p = 0.656
Intensive care units	196	66 (33.7)	1.39 (0.94-2.05)	p = 0.103
Emergency Department	67	35 (52.2)	3.16 (1.82-5.45)	p < 0.001
Hospice	12	5 (41.7)	1.79 (0.55-5.82)	p = 0.334
Oncology*	310	83 (26.8)	1.00	
**Next of kin**	646			p < 0.01
Spouse	190	85 (44.7)	1.89 (1.05-3.41)	p < 0.05
Child	329	109 (33.1)	1.04 (0.58-1.85)	p = 0.905
Parents	38	13 (34.2)	2.96 (1.09-8.04)	p < 0.05
Other	12	5 (41.7)	1.63 (0.46-5.85)	p = 0.452
Siblings*	77	21 (27.3)	1.00	
**Way of contacting**	653			p = 0.80
Face-to-face	414	138 (33.3)	1.00 (0.71-1.41)	
Telephone*	239	82 (34.3)	1.00	
**Day of the week**	824			p = 0.039
Sunday	142	35 (24.6)	1.13 (0.62-2.06)	p = 0.691
Monday	115	44 (38.3)	2.12 (1.17-3.86)	p < 0.05
Tuesday	120	34 (28.3)	1.36 (0.74-2.50)	p = 0.331
Wednesday	118	37 (31.4)	1.61 (0.88-2.95)	p = 0.125
Thursday	100	39 (39.0)	2.28 (1.23-4.21)	p < 0.01
Saturday	124	41 (33.1)	1.69 (0.93-3.07)	p = 0.087
Friday*	105	23 (21.9)	1.00	
**Donor card registration –deceased**	824			p < 0.001
Yes	75	58 (77.3)	10.10 (5.84-18.40)	p < 0.001
No*	749	195 (26.0)	1.00	
**Donor card registration – family member**	787			p < 0.001
Yes	274	165 (59.9)	7.67 (5.49-10.80)	p < 0.001
No*	513	83 (16.2)	1.00	
**Combined donor card registration**	787			p < 0.001
Deceased Yes & Family Yes	60	50 (83.3)	29.80 (15.20-65.20)	p < 0.001
Deceased Yes & Family No	12	8 (66.6)	11.20 (3.41-42.90)	p < 0.001
Deceased No & Family Yes	214	114 (53.2)	6.37 (4.44-9.22)	p < 0.001
Deceased No & Family No*	501	75 (15.0)	1.00	

*Referent category. #Adjusted for age and sex of deceased.

### Multivariable analysis

A multivariable logistic regression analysis was conducted on 559 cases with complete data to identify factors independently associated with consent for corneal donation. Cases with missing data were excluded from the analysis. The model included variables that were found significant in the univariate analysis, excluding religiosity, because it was only available among Jewish participants and therefore was analyzed separately. In the final step of the model, older age, donor card registration of either the deceased or a family member and religion were independently associated with higher likelihood of family consent to corneal donation (Table 4). Regarding religion, Muslim religion was used as reference category, Jewish families (OR = 8.32 p < 0.001) and Christian families (OR = 11.7 p < 0.01) had significantly higher odds of consent.

**Table 4:** Multivariable analysis among all patients.

Variable	Adjusted OR (95% CI)	P value
Age	1.02 (1.00-1.04)	p < 0.05
Religion* - Jewish	8.32 (2.96-34.9)	p < 0.001
Religion* – Christian	11.7 (1.83- 77.0)	p < 0.01
Donor card registration –deceased	5.18 (2.61- 11.0)	p < 0.001
Donor card registration –family member	4.95 (3.32–7.46)	p < 0.001

* Muslim religion was used as the reference category

Table 4 provides the outcomes of multivariate analysis among all patients.

A separate logistic regression was performed among Jewish individuals with complete data (n = 366). In the final stepwise model, religiosity, donor card registration among deceased and among first-degree family member remained significant predictors. Using traditional religiosity as the reference category higher odds of consent were observed among secular (OR = 4.74, p < 0.005) and modern Orthodox individuals (OR = 4.51, p < 0.05). Donor card registration was significant among the deceased (OR = 3.35 p < 0.005), as was among family members (OR = 3.81 p < 0.001).

Table 5 represents the results of multivariable analysis among Jewish patients.

**Table 5:** Multivariable analysis among Jewish patients.

Variable	Adjusted OR (95% CI)	P value
Jewish religiosity* - Secular	4.74 (1.73–15.4)	p < 0.005
Jewish religiosity* -Modern Orthodox	4.51 (1.44–16.2)	p < 0.05
Donor card registration –deceased	3.35 (1.95–5.87)	p < 0.005
Donor card registration –family member	3.81 (1.63–10.1)	p < 0.001

* Traditional religiosity was used as the reference category

## Discussion

This study examined the factors affecting family consent for corneal donation among potential donors, focusing on clinical, demographic, situational, and cultural variables. Conducted at the Hadassah Medical Center Eye Bank in Israel, the study is notable for its culturally diverse population and the short time interval between death and the family approach. The overall consent rate of 30.7% highlights substantial opportunity to improve corneal donation through targeted interventions. In multivariable analysis, religion, Jewish religiosity, donor card registration of both the deceased and a first-degree family member, and older age were independently associated with family consent. These findings suggest that family decisions are influenced both by cultural background and by previously expressed donation intentions.

Donor card registration emerged as one of the strongest independent predictors of family consent. Consent increased from 26.0% among unregistered deceased individuals to 77.3% among registered donors (OR = 10.10), reached 59.9% when a first-degree family member was registered (OR = 7.67), and was highest when both the deceased and a family member were registered (83.3%, OR = 29.80). Nevertheless, 17 registered donors did not ultimately become donors because their families declined consent, highlighting the importance of real time access to donor registration information during the family approach.

In the multivariable analysis, using Muslim families as the reference group, Jewish families had substantially higher odds of consent (OR = 8.32, p < 0.001), while Christian families also demonstrated higher odds of consent (OR = 11.7 p < 0.01). These findings should be interpreted with caution because the Christian subgroup was small and donor card registration differed markedly across religious groups. Since donor card registration was a strong independent predictor of consent, adjustment for this variable may have attenuated the estimated association between religion and consent. Larger studies are needed to further clarify these relationships.

Previous Israeli studies of organ donation have similarly reported lower consent rates among Muslim families despite the generally supportive Islamic position toward organ donation, suggesting that donation decisions may be influenced by factors beyond formal religious doctrine, including limited awareness of organ and tissue donation, uncertainty regarding religious ruling, family dynamics, trust in the healthcare system, concerns regarding the sacredness of the body, and the importance of prompt burial [22–24].

Donor card registration was substantially more common among Jewish individuals (11.2%) than among Muslims (0.7%) or Christians (4.8%). These differences may partially explain the variation in consent rates observed between religious groups. However, because both religion and donor card registration remained independently associated with consent in the multivariable model, these findings suggest that cultural background and previously expressed donation intentions capture complementary aspects of family decision making. These observations further support culturally tailored educational initiatives aimed at increasing awareness of corneal donation [25].

Among Jewish families, secular and modern Orthodox groups had the highest consent rates (57.3% and 53.6%, respectively), compared with only 0.9% among ultra-Orthodox families. In the multivariable analysis of the Jewish subgroup, secular and modern Orthodox families remained significantly more likely to consent.

Higher consent rates among secular and modern Orthodox families may reflect more progressive interpretations of religious law and greater acceptance of medical procedures such as organ and tissue donation, whereas the very low consent rate observed among ultra-Orthodox families may be related to ongoing Jewish law debates surrounding organ donation. Given the significant association between religiosity and consent, culturally tailored communication and educational initiatives may help address religious concerns and improve donation rates in more observant communities.

Although older age remained independently associated with family consent in the multivariable model, the magnitude of this association was modest (aOR = 1.02 per year), with a confidence interval close to unity. Furthermore, no significant association was observed when age was analyzed categorically. Therefore, despite its statistical significance, the clinical relevance of age appears limited and should be interpreted cautiously. Similar variability has been reported in previous studies, suggesting that the influence of age on donation decisions is modest and may differ across populations [12].

The identity of the next of kin also influenced family consent. Parents and spouses were more likely to consent than siblings (OR = 2.96 and 1.89, respectively). These findings are consistent with previous studies, which also found that parents were the most likely family members to consent to donation [26].

Interestingly, the method of contact, whether face-to-face or via telephone, was not associated with differences in consent rates (p = 0.80). This finding supports the feasibility of telephone-based approaches, especially when in-person communication is logistically challenging. It aligns with previous studies demonstrating the effectiveness of remote consent processes [27].

The mean interval between death and the family approach was only 1.8 hours, considerably shorter than the 8–13 hours intervals reported in previous studies. [11,28] This reflects the unique cultural and logistical context in Israel, particularly in Jerusalem, where same day burial is customary according to Jewish tradition. The urgency surrounding burial, especially before the Sabbath, may contribute to lower willingness to delay funeral arrangements for tissue donation, which is consistent with the lower consent rates observed on Fridays.

The hospital department at the time of death was associated with consent. Families of patients who died in the Emergency Department (ED) were more likely to consent (OR = 3.16), while those from Internal Medicine departments showed lower consent rates. Although this variable did not remain in the multivariable model, it reflects the importance of situational context. For example, in the ED, families are often physically present and may already be engaged in critical decision-making processes. Furthermore, the sudden and visible nature of death in the ED may facilitate quicker acceptance of death, contributing to higher consent rates. This phenomenon has also been observed in kidney donation following uncontrolled circulatory death (uDCD), where consent rates sometimes exceed those seen in brain death scenarios [29].

Potential donors in the non-consent group accumulated slightly more hospitalization days during the last year of life than those in the consent group (mean difference: 8.7 days). This finding may be consistent with the higher consent rates observed among patients who died in the Emergency Department, where deaths are generally more sudden and prolonged hospitalization is less common. However, this association was no longer significant after multivariable adjustment.

This study has several limitations. First, religiosity was assessed by experienced transplant coordinators as part of routine clinical practice rather than using a validated instrument. Consequently, some degree of misclassification and observer bias is possible. However, because the study focused on socioreligious affiliation as a cultural context for donation related decision making rather than individual religious belief or practice, this pragmatic approach was considered appropriate for the clinical setting. The specific reasons for family consent or refusal were not systematically recorded, and families often provided no explicit or only nonspecific explanations. Ongoing data collection at our eye bank aims to document these reasons more systematically. Furthermore, interpersonal variation among staff was not measured, although it may have influenced consent outcomes. Thirdly, multivariable analyses were restricted to complete cases. Of the 824 eligible cases, 559 (67.8%) were included in the overall multivariable model. For the Jewish subgroup analysis, 366 of 652 cases (56.1%) were included. Missing data were primarily due to missing values in variables such as next-of-kin identity and religiosity. Although key predictors demonstrated significant effect sizes, this complete case approach may have reduced statistical power or introduced potential selection bias. Finally, the study was conducted at a single center, which may limit the generalizability of the findings.

This study examined a broad range of clinical, demographic, and contextual factors associated with family consent for corneal donation. In multivariable analyses, religion, Jewish religiosity, donor card registration of both the deceased and a first-degree family member, and older age were independently associated with family consent. Taken together, these findings suggest that both cultural factors and previously expressed donation intentions play an important role in family decision making. Improving public awareness of corneal donation, increasing donor card registration, providing transplant coordinators with real-time access to donor registration status, and adopting culturally sensitive communication strategies may help increase family consent rates and ultimately improve the availability of corneal tissue for transplantation.
